# How sense-of-direction and learning intentionality relate to spatial knowledge acquisition in the environment

**DOI:** 10.1186/s41235-017-0057-4

**Published:** 2017-03-20

**Authors:** Heather Burte, Daniel R. Montello

**Affiliations:** 1 Department of Psychology, Tufts University, 490 Boston Ave, Medford, MA 02155 USA; 2 Department of Geography, University of California Santa Barbara, 1832 Ellison Hall, Santa Barbara, CA 93106 USA

**Keywords:** Spatial cognition, Spatial knowledge acquisition, Sense-of-direction, Intentional and incidental learning

## Abstract

People’s impression of their own “sense-of-direction” (SOD) is related to their ability to effectively find their way through environments, such as neighborhoods and cities, but is also related to the speed and accuracy with which they learn new environments. In the current literature, it is unclear whether the cognitive skills underlying SOD require intentional cognitive effort to produce accurate knowledge of a new environment. The cognitive skills underlying SOD could exert their influence automatically—without conscious intention—or they might need to be intentionally and effortfully applied. Determining the intentionality of acquiring environmental spatial knowledge would shed light on whether individuals with a poor SOD can be trained to use the skill set of an individual with good SOD, thereby improving their wayfinding and spatial learning. Therefore, this research investigates the accuracy of spatial knowledge acquisition during a walk through a previously unfamiliar neighborhood by individuals with differing levels of self-assessed SOD, as a function of whether their spatial learning was intentional or incidental. After walking a route through the neighborhood, participants completed landmark, route, and survey knowledge tasks. SOD was related to the accuracy of acquired spatial knowledge, as has been found previously. However, learning intentionality did not affect spatial knowledge acquisition, neither as a main effect nor in interaction with SOD. This research reveals that while the accuracy of spatial knowledge acquired via direct travel through an environment is validly measured by self-reported SOD, the spatial skills behind a good SOD appear to operate with or without intentional application.

## Significance

Imagine a friend invites you to a restaurant in a neighborhood familiar to your friend, but unfamiliar to you. During your stroll to the restaurant, you chitchat as you pass Spanish-style homes with manicured lawns and wander through a natural park. Upon arriving at the restaurant, your friend gets an emergency call and must leave immediately. Can you find your way home alone? Your success in returning home likely depends on two factors: your sense-of-direction (SOD) and your intention to learn the route along the way. If you have a good SOD and/or were intentionally noting the layout of the neighborhood, then you might be very successful in returning home unaided. In contrast, if you have a poor SOD and/or were absorbed in the conversation, then you might be completely unable to return home by yourself. This research aims to answer the question: Is it the case that people with good environmental spatial skills express that skill only when they intentionally pay attention to an environment or does their greater skill express itself without intentional effort? In this research, participants walked a route while either gathering impressions of architecture (incidental learning) or intentionally learning the spatial layout of an unfamiliar neighborhood. They then completed spatial tasks—mimicking the restaurant scenario. SOD, but not learning condition, reliably predicted spatial knowledge, indicating that the spatial skills associated with SOD express themselves regardless of one’s intentionality to learn the spatial layout of a novel environment.

## Background

The ability to learn spatial properties of novel environments is an important aspect of our everyday lives—whether learning a new city you moved to or navigating through an unfamiliar airport. Spatial knowledge acquisition includes learning both metric and non-metric spatial properties of environments; metric properties include quantitative distances and directions and non-metric properties include sequence and connectivity. These spatial properties include the identities and locations of landmarks, the turns in a route, and the distances and directions between places (Goldin & Thorndyke, 1982; Thorndyke & Hayes-Roth, 1982). Environments can be learned directly by people sensing and moving through the environment or indirectly via symbolic sources of information, such as maps or language (Montello & Freundschuh, 1995). And of course, individuals differ in how well and how easily they learn spatial knowledge about environments. But an interesting and important question remains: Do people with good environmental spatial skills express that skill only when they intentionally pay attention to spatial properties or does their greater skill express itself without intentional effort? In this research, we examine how the individual-difference trait of “sense-of-direction” (SOD) expresses itself when people directly learn spatial properties of a new neighborhood. We specifically examine this relationship as a function of whether people receive intentional or incidental instructions to learn the spatial properties.

### Directly acquiring spatial knowledge in the environment

Two accounts have been proposed to explain the process of acquiring spatial knowledge in new environments (microgenesis) from direct experience, without symbolic sources: the “dominant framework” proposed by Siegel and White (1975) and an “alternative framework” proposed by Montello (1998). Siegel and White (1975) contended that the learning of a new environment progressed through three hierarchical “stages.” First, individuals learn landmarks, which are salient point-like structures that lack distance and directional information (i.e., non-spatial identity). At this stage, landmark knowledge consists of familiarity with the appearance and perhaps the names of the landmarks, but no knowledge of how those landmarks are spatially related (assuming they are out of sight from each other). After acquiring a sufficient level of landmark knowledge, individuals learn the route(s) that connect the landmarks into a sequence of movements (non-metric only). For example, route knowledge might include knowing that landmark A comes before landmark B along a specific route and that at landmark A one needs to turn right to get to landmark B. Finally, after acquiring a sufficient number of routes and beginning to metrically scale them, individuals relate the landmarks and routes to one another as part of a metric spatial configuration, referred to as survey knowledge. The strict hierarchical nature of these stages meant that individuals could not have survey knowledge without first passing through landmark knowledge and then route knowledge. With only landmark and route knowledge (at least in its initial form), a person would not have accurate metric knowledge of environmental layout, such as knowledge of direct distances and directions between landmarks.

In contrast to what Montello (1998) termed the dominant framework, he proposed an alternative framework in which individuals continuously acquire all three forms of knowledge from the beginning of a single episode traveling in an environment, without the need to pass from one stage to another. For example, most people have probably experienced walking in an unfamiliar city for 30 min and knowing more than just the identities of landmarks they have passed—most would have some ability to retrace a route back to their starting location. Many people could probably estimate distances and directions towards at least some of the landmarks they passed along the route—albeit not very precisely or perfectly accurately after only one travel experience. Thus, it is likely that people acquire some level of route and survey knowledge after just minutes of exposure to a new environment and a single travel episode. The alternative framework contends that landmark, route, and survey knowledge are acquired more or less simultaneously, as soon as an individual starts experiencing a new environment. Of course, the accuracy and completeness of this knowledge increases with experience, potentially indefinitely.

In the current study, participants were taken on a walk through an unfamiliar housing development. If the dominant framework accurately describes spatial microgenesis, we should not expect any participants to acquire much metric knowledge about distances and directions; it is questionable if they would even acquire much information about sequences of places along routes. However, if the alternative framework more accurately describes spatial microgenesis, we expect that participants will acquire not only route knowledge but some metric survey knowledge. Only in the latter case should we expect a substantial relationship between the accuracy with which participants estimate metric spatial properties and their SOD.

### Individual differences in acquiring spatial knowledge in the environment

Regardless of which framework describes spatial knowledge acquisition better, one would not expect all individuals to acquire knowledge at the same speed, with the same accuracy, and so on, even if they had similar levels of experience in the environment. While this is an active research topic (Wolbers & Hegarty, 2010), we presume that individuals differ in acquiring knowledge due to some combination of innate or learned spatial abilities and/or acquired strategies for learning and estimating spatial properties. In fact, Ishikawa and Montello (2006) found that individuals, who were driven along a novel route once a week for ten weeks, showed radically different patterns of spatial knowledge acquisition. These differences were particularly salient in their survey knowledge; even though some participants did not acquire accurate survey knowledge after ten trips, these participants did learn the identities of landmarks, the order of landmarks along two test routes, and distances between landmarks along the routes. In essence, even though that study was designed to compare the dominant and alternative frameworks, in the end it showed that individual differences were so substantial that no single framework was likely to describe the learning process well for everyone. Some people’s learning seemed best characterized by the dominant framework, others by the alternative. Some did not show much learning at all over the ten weeks. This finding suggests that we should see considerable variance among our participants in their acquisition of metric survey knowledge, which in turn implies ample variance to support sizeable correlations with self-reported sense-of-direction.

### Cognitive effort and intention to learn

There is a long history of research on the role of intention and effort in learning different kinds of information (Craik & Lockhart, 1972; Hasher & Zacks, 1979; Postman, 1964), some of which has focused on learning spatial information (Mandler, Seegmiller, & Day, 1977; Naveh-Benjamin, 1987). Research on the impact of active versus passive exploration of environments while acquiring spatial knowledge has been mixed (for a review, see Chrastil & Warren, 2012). Lindberg and Gärling (1983) found no differences in survey knowledge after incidental or intentional learning across three exposures to the environment. However, all participants showed performance increases across the three exposures, suggesting that all participants were attending to the spatial properties of the environment. More recently, Van Asselen, Fritschy, and Postma (2006) investigated learning differences within a building and found that landmark identification and ordering did not differ between the incidental and intentional learners. Participants who learned intentionally were more accurate in retracing the route they learned and drawing the route on a map. In a similar study focused on landmark knowledge, landmark identification again did not differ between learning conditions but landmark placement on a map showed a benefit of intentional learning (Wenczel, Hepperle, & von Stülpnagel, 2016), but another study failed to replicate these findings (Von Stülpnagel & Steffens, 2013). Overall, these findings suggest that landmark knowledge and route knowledge might be relatively effortless to acquire, whereas survey knowledge might be more effortful. That is, the level of effortful processing required to learn the spatial properties of an environment might depend on the type of spatial knowledge being acquired. To investigate this possibility, the current research will assess participants’ landmark, route, and survey knowledge after incidental or intentional learning of a novel environment.

A classic method to determine whether a cognitive process requires automatic versus effortful processing is to manipulate the intention to learn (Hasher & Zacks, 1979). This is typically done by instructing some participants to try to learn a certain type of information and not instructing others. If performance is more accurate after intentional than incidental instructions, one can conclude that processing the information requires conscious attention and explicit processing. In contrast, if there are no performance differences whether learning was intentional or incidental, then one can conclude that the cognitive process must be automatic. In the current research, we apply this logic to investigate the interplay between spatial learning intentionality and individual differences in acquiring environmental spatial knowledge from direct experience. Importantly, we will contrast intentional with incidental learning of spatial aspects of the environment (e.g., landmark, route, and survey knowledge) but will not contrast intentional with incidental attention to the environment per se. This was done to mimic the restaurant scenario (see the Significance statement) in which an individual is looking around and attending to the environment but not attending to the spatial properties of the environment (e.g., incidental spatial learning). We will accomplish this by instructing all participants to attend to the environment (using a cover story about attitudes toward architectural and natural features) but instructing only half of the participants that they must learn the spatial layout of the environment and will be tested on it (i.e., intentional spatial learning). By manipulating intentionality in this way, we ensure that all participants are attending to the environment but the groups differ in their intentionality to learn spatial properties.

### Sense-of-direction (SOD)

Ishikawa and Montello (2006) reported that the accuracy and speed with which survey knowledge was acquired by participants were strongly related to their self-reported SOD. SOD is the hypothesized ability to find your way within environmental-scale spaces. It has primarily been assessed by self-report measures, such as by answering the simple question “How good is your sense-of-direction?” (Kozlowski & Bryant, 1977) or by averaging responses to several questions, such as questions about getting lost, learning distances and directions, using maps, and following cardinal directions. Using the multi-item self-report survey known as the Santa Barbara Sense-of-Direction (SBSOD) scale (Hegarty, Richardson, Montello, Lovelace, & Subbiah, 2002), Ishikawa and Montello found that SBSOD scores related mostly to how well participants learned survey relations, such as straight-line directions between landmarks on their test routes. Those who reported having a good SOD learned survey knowledge substantially more accurately and quickly; those who reported having a poor SOD learned them less accurately and quickly, in some cases, virtually failed to learn them at all. In contrast, participants differed very little in their ability to acquire landmark and route knowledge as a function of their SBSOD score; all individuals—regardless of their reported SOD—were able to accurately order named landmarks after one exposure to the route. In fact, most participants were able to accurately estimate distances between landmarks along the route after only one trip, even if they reported a poor SOD. In the current study, we assessed several types of spatial knowledge and related participants’ performance to their self-reported SOD.

Previous research has rarely examined different types of spatial knowledge when examining individual differences in environmental spatial knowledge (e.g., Fields & Shelton, 2006; Hegarty, Montello, Richardson, Ishikawa, & Lovelace, 2006; Montello & Pick, 1993; Schinazi, Nardi, Newcombe, Shipley, & Epstein, 2013), but when multiple measures of spatial knowledge have been related to self-reported SOD, some measures of spatial knowledge relate to SOD and others do not. In the Ishikawa and Montello study, participants easily acquired accurate knowledge of landmark identities and routes, including metric distances along the routes, and these measures were not related to SOD. For their measure of landmark knowledge, Ishikawa and Montello used four landmarks per route (a total of eight for two routes) and they taught participants verbal labels for the landmarks. Naming landmarks could have introduced verbal processing into the processing of spatial information, which might have drawn upon cognitive skills that individuals with poor SOD are not particularly poor at. Support for this idea comes from dual-task paradigms in which verbal tasks interfere with aspects of landmark, route, and survey knowledge (Labate, Pazzaglia, & Hegarty, 2014; Saucier, Bowman, & Elias, 2003; Wen, Ishikawa, & Sato, 2011). In order to address this issue, the current experiment used eight landmarks along a route and the experimenter did not associate the landmark with verbal labels. Instead, the experimenter referred to the landmark scenes by using photographs of each landmark when testing participants’ spatial knowledge. This ensured that while participants may have associated the landmarks with verbal labels, those verbal labels were unique to each participant and not influenced by any verbal label given by the experimenter.

### Interaction between cognitive effort and SOD

The main purpose of the current study is to investigate whether SOD relates to the acquisition of environmental spatial knowledge differently as a function of learning intentionality. This is important because it addresses the question of whether the skills associated with having a good SOD are better characterized as mental abilities (such as memory capacity or mental processing speed) or as strategies (such as paying attention to turns you take or watching the sun as you walk). Mental abilities would typically express themselves implicitly whether a person attempts to apply them or not—they do not require conscious effort to influence knowledge processing. Strategies, on the other hand, can be consciously retrieved by a spatial thinker and accurately described to another person (such as to a researcher during a protocol analysis). Even as a strategy becomes easier to apply with repeated use, people choose to use it when they are trying to solve a particular problem for which they think it is relevant. Note that the distinction here between implicit and explicit does not map perfectly onto the learned-innate distinction. Strategies are presumably learned, but mental abilities may be innate, learned, or (most likely) result from an interaction of innate and learning influences.

The question of how SOD skills relate to learning effort and automaticity is not only theoretically important but is also relevant to the prospect of training people to have a better SOD. If SOD skills are due to explicitly applied strategies for spatial problem-solving, then it will likely be easier and more straightforward to train individuals for better skill (e.g., Hegarty, Keehner, Cohen, Montello, & Lippa, 2007; Thorndyke & Stasz, 1980). It may still be possible to improve mental abilities expressed without conscious application, however, given appropriate training experiences (cf. Uttal et al., 2013). This may be true even for innate abilities; innate does not mean unchangeable, although it would typically mean less easily changeable. We expect that training mental abilities would be considerably less straightforward than simply telling people to use a specific strategy while solving a problem.

In sum, if SOD reflects learned strategies under conscious control, we should find at least a modest main effect of spatial learning intentionality on spatial knowledge acquisition, because people with good SOD would learn better under intentional instructions than incidental (it is unclear if people with poor SOD could learn the spatial layout better when intentionally attending to spatial properties of the environment or not). In particular, however, we should find an interaction between learning intentionality and SOD, because individual differences in spatial knowledge acquisition would be diminished when the spatial layout of an environment was experienced incidentally, without the intention to learn it. When people with varying SODs are not told to pay attention to spatial properties (incidental learning), they would not be as likely to apply their strategies and thus would not differ much in the spatial knowledge they acquire. When the spatial layout of an environment is experienced intentionally, however, individuals with good SOD would learn spatial aspects of environments better because they would better apply particular strategies to learning when told to do so. Therefore, we would expect that we might find a main effect of learning intentionality but that we would definitely find an interaction between SOD and learning intentionality.

In contrast, if SOD is a reflection of an individual’s implicit mental ability to acquire spatial knowledge, then it should not matter whether instructions to learn spatial properties are intentional or incidental (i.e., learning intentionality would have no effect). Those with good SOD would learn more and more accurately whatever the instructions they receive. It could even be that what underlies a good SOD is the unprovoked tendency to attend to spatial properties without being told to do so. That is, maybe people with a good SOD are always attending to the spatial properties of an environment, even when only given incidental instructions. Those with a poor SOD never or rarely attend to spatial properties, so even when told to attend to spatial properties, they do not know how to or they cannot make use of the spatial properties they notice. Whatever the nature of the implicit abilities underlying SOD, we would expect people with good SOD to learn better than those with poor SOD whether they receive incidental or intentional instructions. That is, we expect a main effect of SOD, no main effect of learning intentionality, and no interaction between SOD and learning intentionality.

In the current research, we investigate how environmental spatial knowledge that is acquired directly is related to: (1) SOD; (2) learning intentionality; and (3) the interaction between SOD and learning intentionality.

## Prescreening Methods

We first screened students from a campus research pool to identify a group suitable to participate in our main study. We selected students aged at least 18 years, who were unfamiliar with the experiment location, and either relatively good or poor in self-reported SOD.

### Participants

Across multiple undergraduate courses, 637 students (316 women, 318 men, and three unspecified gender) completed the prescreening questionnaire (Table 1). Enrollment in the courses was over 1000, meaning that approximately 60% of course students completed the prescreening.

**Table 1:** Demographics for prescreening and experiment participants

Measure	Prescreening participants	Eligible participants	Experiment participants	
			Poor SOD	Good SOD
*N*	637	291	42	36
Women	316	156	30	14
Men	318	134	12	22
Unknown	3	1	-	-
Experiment location familiarity	2.9	1.8	2.0	1.9
Mean age	19.3	19.2	18.9	20.0
Age range	17–32	18–28	18–23	18–26
Age standard deviation	1.7	1.6	1.3	2.2
Mean SBSOD	4.8	4.6	3.7	5.9
SBSOD range	1.5–7.0	1.5–7.0	2.1–4.7	5.4–6.9
SBSOD Standard deviation	1.0	1.2	0.7	0.3

### Materials and procedure

We chose an area near campus known as Storke Ranch to be the site for our experiment. It is a condo and single-home neighborhood near the University of California-Santa Barbara (UCSB) campus. The layout of the neighborhood is unconventional as a suburban housing development due to winding streets, a park area that bisects the neighborhood, and multiple cul-de-sacs. Storke Ranch is closed off to surrounding neighborhoods by tall concrete fences and the two-story homes block most visual access to distant spatial referents, such as the mountains to the north. Thus, Storke Ranch provided an adequately complex environment for learning, with few distant spatial referents, but was easily accessible from the UCSB campus. Despite its proximity, Storke Ranch was unfamiliar to most undergraduate students, as it was a new development at the time of the study and did not generally rent to undergraduates.

Potential participants were informed, during lectures or labs, that there was an opportunity to participate in research on “attitudes towards architectural and natural features.” In order to be eligible to participate in the research, they needed to complete the prescreening questionnaire and an experimenter would contact them by email to arrange their participation in the research at a later date. Potential participants completed the questionnaire during or after their lecture or lab and returned the questionnaires to the experimenter.

The prescreening questionnaire consisted of demographics (age and gender), the SBSOD scale (Hegarty et al., 2006), and a familiarity task. The familiarity task involved a labeled map of the UCSB campus and surrounding neighborhoods, divided into 12 areas labeled only as “Area A” through “Area L.” Participants rated their familiarity with each of the 12 areas using a 7-point rating scale, where 7 was “very familiar” and 1 was “not at all familiar.” Although we were interested only in Storke Ranch (labeled “Area B”), we asked about a much wider set of several areas to avoid tipping off potential participants to the experimental location. One to two weeks after completing the prescreening experiment, eligible participants were emailed about participating in the “attitudes towards architectural and natural features” research. Participants completed the experiment typically two to four weeks after being contacted. Participants were not told how or why they were selected and multiple weeks passed between completing the prescreening and experiment.

## Prescreening Results

### Selecting experiment participants

**Fig. 1:**
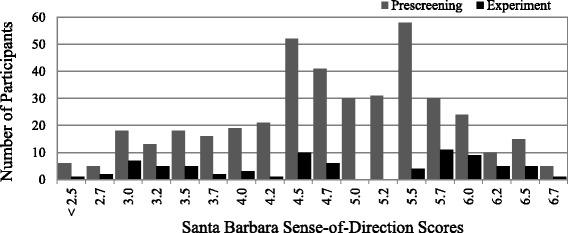
*Histogram* of SBSOD scores for prescreening participants (aged 18 years and unfamiliar with experiment location) and participants who completed the experiment

We preselected participants for participation in this study based on their unfamiliarity with the experiment location (to reduce familiarity effects) and their SOD scores. Preselection based on SOD scores allowed us to ensure that participants in the instruction groups had similar SOD levels and allowed us to compare SOD extremes. Many studies of SOD and similar continuous variables rely on median splits. However, this results in two groups in which very similar participants (those near the split) are separated into different categories. This can be especially problematic in skewed distributions, such as we find with SOD scores (Fig. 1). To reduce this problem, we opted to split eligible participants into three groups based on SOD (good, moderate, and poor) and to include only good and poor SOD participants in our main experiment.

Of the 637 prescreen participants, 635 were aged at least 18 years. Of these, 416 were suitably unfamiliar with the experiment location, Storke Ranch (ratings of 1–3 out of 7); their mean familiarity rating was 1.9. Scores on the SBSOD (*M* = 4.7; *SD* = 1.0) were used to group these students into those with good or poor SOD (*N* = 291), who would be eligible for the main experiment, or those with intermediate SOD (*N* = 125), who would not be (Fig. 1). A group of 104 students (top 25%) were classified as good SOD, operationalized as having a score of 5.5 or greater (*M* = 5.9). A second group of 187 students (bottom 45%) were classified as poor SOD, operationalized as having a score of 4.7 or lower (*M* = 3.7). That left 125 students (middle 30%) with intermediate scores, who were not eligible for the experiment. These groups are summarized in Table 1.

A mean SBSOD score as high as 4.7 may seem rather high for a group to be considered poor on a self-assessed skill on a 7-point scale. In fact, one normally finds mean scores above the midpoint of 4.0 in samples of SBSOD scores (e.g., Montello & Xiao, 2011). Also, using a stricter cutoff for poor SBSOD left us with too few of those participants willing to participate in the experiment. Therefore, we used the slightly higher cutoff.

### Gender effects

Among the 634 prescreening participants who reported their gender, men reported significantly higher SBSOD scores (*M* = 5.1) compared to women (*M* = 4.5), *t*(632) = –7.22, *p* < 0.001. Of the participants in the main experiment, 71% of the poor SOD participants and 39% of the good SOD participants were women. This gender difference in the makeup of the SOD groups is statistically significant, *χ* ^*2*^(1) = 8.35, *p* < 0.01.

## Main Experiment Methods

### Participants

All 291 eligible participants were contacted to participate in the experiment. Eighty students in total participated but, due to a typographical error, that included two moderate SOD students. These students were incorrectly labeled as having a good SOD, so both were excluded from analyses. This resulted in 36 good and 42 poor SOD students participating in the experiment. These participants were randomly assigned to a learning condition such that 17 good and 21 poor SOD participants learned intentionally and 19 good and 21 poor SOD participants learned incidentally. Those who participated in the experiment were compensated with course credit.

### Materials

**Fig. 2:**
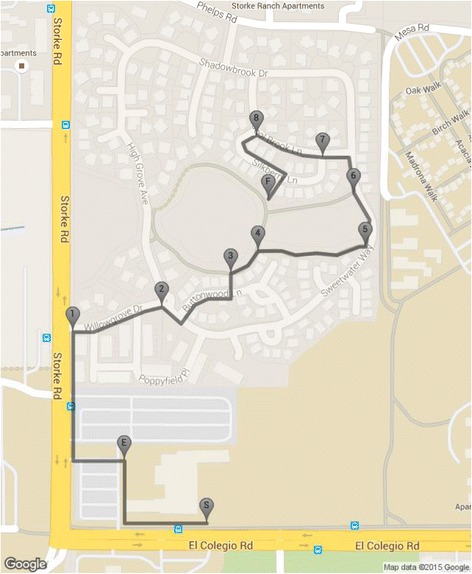
Google Map™ of Storke Ranch with the actual route traveled in *dark gray* and stop-points. *S* start, *E* example stop-point, *F* finish and testing location

As described previously, the Storke Ranch neighborhood provided the site for our experiment (Fig. 2). We designed the 1.28-km route to be walkable within 15 min and moderately complex, with numerous recognizable landmarks so that route locations could be recognized from photographs. The start of the route was just outside Storke Ranch, at the off-campus Santa Catalina dormitory, where many freshman students resided and were familiar with (mean familiarity with Santa Catalina was 3.3/7 for prescreening participants).

**Fig. 3:**
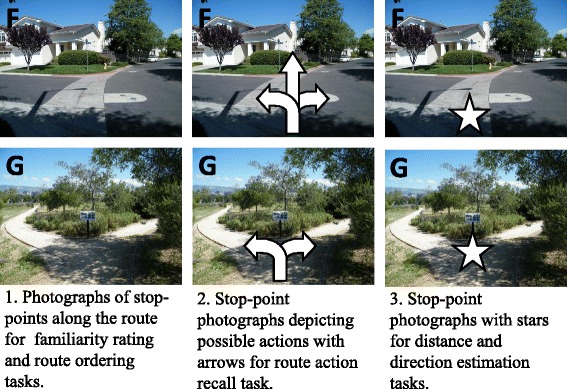
Stop-point photographs when used for the landmark recognition task (*left*), route ordering task (*middle*), and for distance and direction estimation (*right*). Stop-points were labeled with letters: F is the seventh stop-point and G is the fourth stop-point

Participants were stopped at nine “stop-points” along the route and asked to attend to the “important area” in front of them. To avoid the experimenter associating stop-points with verbal cues that might draw participants’ attention to a particular feature at the location (spatial or architectural), the experimenter did not say anything about the stop-point when stopped at the location during the learning phase. When showing the participants photographs of views at the stop-points during the testing phase, the experimenter only used the letter assigned to each stop-point photograph. Stop-point photographs were labeled with a letter A–H, which was randomly assigned so that the letter sequence did not correspond with the route sequence of stop-points. The stop-point photographs were taken from the same perspective the participant saw while walking the route (i.e., direction of travel) and were taken around 10:00–12:00, so that shadows were minimized (examples of stop-point photos are shown in Fig. 3). Distances and directions between stop-points and the testing location at the end of the route were determined using Global Positioning System (GPS).

The testing phase consisted of five tasks: landmark familiarity rating to assess landmark knowledge, stop-point ordering and action recall to assess route knowledge, and distance and direction estimation to assess survey knowledge. Participants were also asked three questions about what they attended to along the route, how effectively they learned the spatial layout of the neighborhood (and if not effective, what they would have changed), and what aspects of a neighborhood are important to pay attention to in order to become familiar with and travel efficiently through it. These questions were included to look for group differences but participant responses did not provide much insights due to the brevity of their responses. We could only confirm that incidental participants felt that they were ineffective at learning the spatial layout and would have “paid more attention.”

#### Stop-point familiarity

Participants viewed stop-point photographs and rated their familiarity with the location on a 7-point scale, with “7” indicating they were very familiar with the stop-point location and “1” indicating that they were not at all familiar. Subjective familiarity ratings were used to capture variation in participants’ familiarity with a location, as opposed to an objective recognition task that do not capture variation in certainty. This measure was also used to determine whether or not group differences in the other spatial measures might have been due to differences in familiarity.

#### Stop-point ordering

All the stop-point photographs were presented simultaneously to participants and participants used the letters associated with each stop-point photograph to indicate the order they recalled experiencing the stop-point locations along the route.

#### Stop-point action recall

Participants viewed stop-point photographs with arrows superimposed onto them and then indicated which of three possible actions they recalled taking at each stop-point (continued on course, turned right, or turned left) (Fig. 3).

#### Direction estimates

Participants estimated the direction from the testing location to the location indicated by a star superimposed on the stop-point photographs by drawing an arrow in a circle (Fig. 3); they actually faced 51° west from true north as they estimated this direction. Relative to the participant’s heading during test, the range of bearings to the stop-points was 40° to 284° (i.e., 11° to 127° west of true north).

#### Distance estimates

Straight-line or “as the crow flies” distance estimates were collected using the psychophysical technique of magnitude estimation, in which participants are shown a standard unit of distance and estimate test distances by providing a number to represent the distance relative to the standard. In our study, participants estimated the distance from their physical location to each stop-point location (specifically from the test location to the location of the superimposed star in each stop-point photograph). The standard was the perceived distance from their physical location to a fence located directly in front of them. The standard was 20 m, although we did not inform participants of this value. Participants were instructed to treat the standard as one unit of distance and to estimate distances towards the stop-point locations to the nearest half-standard unit. Actual distances from the testing location to the stop-points ranged from 65 m (3.2 units) to 327 m (16.2 units).

### Procedure

Participants were randomly assigned to either the incidental or intentional learning condition. Matching was used to balance the number of good and poor SOD students within each learning condition; however, the experimenter was blind to the SOD of the participants during testing. Participants first completed the learning phase by walking the route through the environment with the experimenter; they then completed the testing phase.

#### Learning phase

The experimenter met participants individually outside the Santa Catalina dorms to complete research on “attitudes towards architectural and natural features.” Participants were told they would accompany the experimenter on a walk through the nearby Storke Ranch neighborhood. Specifically, they were told to “focus on noting the appearance of the neighborhood, in terms of architecture, overall design of the neighborhood, and any salient features. During the walk, [the experimenter] will stop you at important areas for you to note the architectural and/or natural features at that location. After our walk, you will answer questions about your impressions of the neighborhood.” All participants were given these instructions; thus, the incidental condition was designed to be incidental only with respect to learning the spatial layout of the neighborhood. It was not incidental with respect to paying attention to the appearance of the architecture, etc.

If the participant was in the intentional learning condition, the experimenter also told the participant to “focus on learning the spatial configuration of the neighborhood. You need to learn what is in the neighborhood, and also how the neighborhood is laid out, which includes how parts of the neighborhood are connected to each other. You will be tested on how well you know the route we walk, as well as the distances and directions between locations.” Other than this instructional difference, there were no differences between the two learning conditions. In sum, the learning conditions differed only in the instructions given to participants about their intentionality to learn the spatial layout of the environment.

The walk proceeded. At each stop-point, the experimenter would stop, state that the location was an “important location,” pause for 5 s, and continue walking. This allowed participants enough time to study the stop-points, so they would be familiar with them during testing. The experimenter and participant spoke while on the walk, but the experimenter ensured that the conversations were light and unrelated to the study. These conversations would cease when they reached a stop-point. The experimenter walked at a quick but relaxed pace; however, the participant’s own natural walking speed was a factor in setting the pace. The route took 15–20 min to walk.

#### Testing phase

At the end of the route, participants were informed that the research was not focused on architectural and natural features, but actually on how people learn new environments. Participants in the incidental condition were surprised by this revelation and frequently remarked that did not think they would perform well on the spatial tasks. All participants completed the testing tasks in the following order: landmark familiarity rating, stop-point ordering, action recall, distance estimation, and direction estimation. All tasks used the stop-point photographs to refer to places along the route; they were presented to each participant in the same order, an order that we determined randomly. None of the participants in the incidental learning condition informed the researcher, nor indicated in their answers to questions, that they knew the experiment was about spatial learning or was not about attitudes concerning architectural and natural features. Most of the incidental learners reported that they were “not effective” in learning the spatial layout of the environment and would have “paid more attention” if they had known that they were going to be tested on the spatial properties of the environment.

## Main Experiment Results

### Power analysis

Previous studies have consistently found non-significant learning condition effects (intentional versus incidental learners) for landmark and route tasks, and have inconsistently found significant learning condition effects for survey tasks (Van Asselen et al., 2006; Von Stülpnagel & Steffens, 2013; Wenczel et al., 2016). Using the summary statistics reported in those studies, we calculated effect sizes for both types of tasks separately. Landmark and route task effect sizes were in the range of 0.0–0.58 (*M* = 0.24) while survey task effect sizes were in the range of 0.69–1.12 (*M* = 0.87). Given these effect sizes and a t-test comparison of learning condition, our power to detect landmark and route task differences was 0.18 and our power to detect survey task differences was 0.97. This indicates that our study is under powered to detect small landmark and route task differences (because no significant differences have been found in previous studies) but sufficiently powered to detect large survey task differences between the two learning conditions.

### Multivariate analysis of variance (MANOVA)

Given that we preselected participants based on their SOD scores and grouped them by SOD, we modeled SOD as a discrete variable. We do not want to force a linear interpolation onto moderate SOD participants who are not represented within our study. If we modeled SOD as a continuous variable (which likely characterizes it accurately as an individual-difference variable), we would be assuming that performance of moderate SOD participants falls linearly between the performance of poor and good SOD participants. Since we do not know this to be the case, we modeled SOD as a discrete variable.

A 2 SOD group (poor, good) by 2 learning condition (incidental, intentional) MANOVA with gender (female, male) was run on the five dependent measures together: average stop-point familiarity (range of 1–7, with 7 being “very familiar”), ordinal route error (range of 0–28, with 0 meaning all stop-points were perfectly ordered), correctly recalled route actions (range of 0–8, with 8 meaning all stop-point actions were recalled correctly), average absolute directional error (range of 0–180°), and distance correlation (range of 0–1 with 1 meaning a perfect linear relationship existed between the participant’s distance estimates and the actual distances between stop-points). More details about how each dependent measure was calculated are provided in the presentation of univariate analyses below and Table 2 reports the means and standard deviations for each measure. The main effect of SOD group was significant, *F*(5, 69) = 5.67, *p* < 0.001, *η* ^*2*^ = 0.26, but the main effect of learning condition, *F*(5, 69) = 0.85, *p* = 0.52, *η* ^*2*^ = 0.05, the main effect of gender, *F*(5, 69) = 0.72, *p* = 0.61, *η* ^*2*^ = 0.05, and the interaction of SOD and learning condition were not significant, *F*(5, 69) = 0.62, *p* = 0.69, *η* ^*2*^ = 0.04. Across all dependent measures, differences in SOD resulted in significantly different knowledge of the spatial layout of the environment. The intentionality of learning instructions did not matter and this lack of influence was equally true for both SOD groups. To explore these results more closely, we next look at each measure of spatial learning individually.

**Table 2:** Means and standard deviations for each measure of spatial knowledge

	Poor SOD		Good SOD		Incidental learning		Intentional learning	
	Mean	SD	Mean	SD	Mean	SD	Mean	SD
Average familiarity	5.1	0.9	6.1	0.8	5.5	0.9	5.7	1.0
Ordinal route error	3.7	3.5	1.8	2.7	3.4	3.5	2.2	3.0
Correct route action	6.6	1.1	7.1	1.1	6.8	1.1	6.8	1.1
Absolute directional error	56°	25°	43°	20°	54°	26°	46°	20°
Distance correlation	0.58	0.45	0.69	0.53	0.62	0.42	0.65	0.56
	Incidental poor SOD		Intentional poor SOD		Incidental good SOD		Intentional good SOD	
	Mean	SD	Mean	SD	Mean	SD	Mean	SD
Average familiarity	5.1	0.9	5.2	0.9	5.9	0.8	6.2	0.7
Ordinal route error	4.6	4.1	2.9	2.7	2.0	2.2	1.5	3.2
Correct route action	6.7	1.2	6.4	1.0	6.9	1.0	7.3	1.1
Absolute directional error	61°	30°	51°	19°	46°	19°	42°	21°
Distance correlation	0.55	0.47	0.60	0.43	0.68	0.33	0.71	0.65

### Stop-point familiarity

**Fig. 4:**
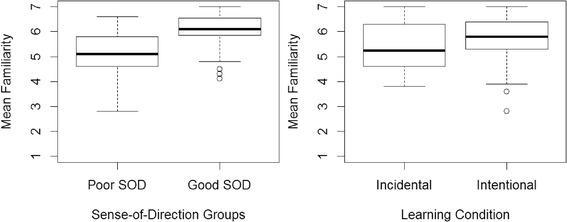
Mean familiarity by SOD group and learning condition. *Center* of the box represents the mean, the *top* and *bottom* of the box indicate the first and third quartile, the *whiskers* indicate a 95% confidence interval, and the *circles* outside the whiskers represent outliers

Average self-reported familiarity for the eight stop-points (range of 1–7, with 7 being “very familiar”) was high at 5.6 (*SD* = 1.0), indicating that participants generally felt quite familiar with the stop-points. A 2 SOD group (poor, good) by 2 learning condition (incidental, intentional) ANOVA found a significant main effect of SOD group, *F*(1, 73) = 22.00, *p* < 0.001, *η* ^*2*^ = 0.22. As suggested by Fig. 4, participants with a good SOD reported greater familiarity with the stop-points than did participants with a poor SOD. The main effect of learning condition, *F*(1,73) = 1.60, *p* = 0.21, *η* ^*2*^ = 0.02, the main effect of gender, *F*(1,73) = 0.05, *p* = 0.83, *η* ^*2*^ < 0.01, and the interaction of SOD and learning condition were not significant, *F*(1, 73) = 0.33, *p* = 0.57, *η* ^*2*^ < 0.01. This result confirmed that the learning conditions did not differ in familiarity; therefore, any differences between the learning conditions could not be attributed to simply differences in familiarity.

### Stop-point ordering

We calculated an ordinal route error measure to quantify the error participants made in the stop-point ordering task as compared to the actual stop-point order (e.g., A then B then C). Every pair of locations (e.g., A and B, or B and C) was assessed independently in terms of whether that pair was placed in the correct order respective to how the two locations were encountered along the route (e.g., for the A-B pair, A came before B along the route). If a participant’s recalled sequence had a pair in the right order (e.g., for the A-B pair, A was ordered before B), that pair was given a 0. If the participant recalled a pair in the wrong order, that pair was given a 1 (e.g., for the A-B pair, A was ordered after B). Given there were eight locations to recall, there were a combination of 28 pairs to assess in this way. Thus, stop-point orders could be scored from 0 to 28; a sequence ordered perfectly would receive a score of 0 and a sequence ordered as poorly as possible would receive a score of 28.

**Fig. 5:**
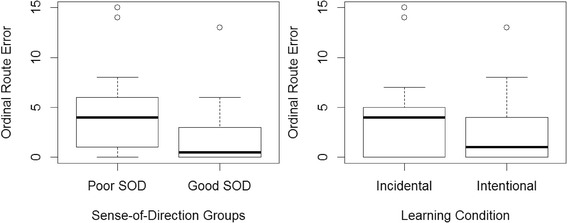
Ordinal route error by SOD group and learning condition. *Center* of the box represents the mean, the *top* and *bottom* of the box indicate the first and third quartile, the *whiskers* indicate a 95% confidence interval, and the *circles* outside the whiskers represent outliers

The mean ordinal route error was quite low at 2.8 (*SD* = 3.3), indicating that on average participants incorrectly ordered 3/28 pairs. A 2 SOD group (poor, good) by 2 learning condition (incidental, intentional) ANOVA found a significant main effect of SOD group, *F*(1, 73) = 7.42, *p* < 0.01, *η* ^*2*^ = 0.10. As suggested by Fig. 5, participants with a good SOD made slightly fewer ordering errors than did poor SOD participants. The main effect of learning condition, *F*(1, 73) = 2.64, *p* = 0.11, *η* ^*2*^ = 0.05, the main effect of gender, *F*(1,73) = 0.15, *p* = 0.70, *η* ^*2*^ < 0.01, and the interaction of SOD and learning condition were not significant, *F*(1, 73) = 0.70, *p* = 0.40, *η* ^*2*^ < 0.01.

### Stop-point action recall

**Fig. 6:**
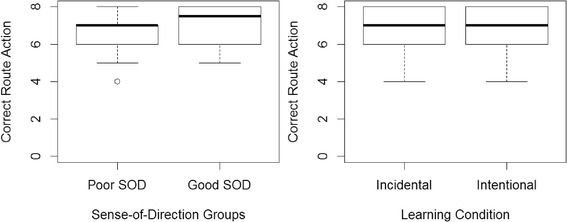
Correct route action by SOD group and learning condition. *Center* of the box represents the mean, the *top* and *bottom* of the box indicate the first and third quartile, the *whiskers* indicate a 95% confidence interval, and the *circles* outside the whiskers represent outliers

The number of correctly recalled stop-point actions was calculated for the eight stop-points (range 0–8, with 8 meaning that all actions were recalled correctly). The mean correct route actions recalled by participants was 6.8 (*SD* = 1.1), indicating that participants were very accurate in recalling the actions they took at the stop-points. A 2 SOD group (poor, good) by 2 learning condition (incidental, intentional) ANOVA found a significant main effect of SOD group, *F*(1, 73) = 4.77, *p* < 0.05, *η* ^*2*^ = 0.04. As suggested by Fig. 6, participants with a good SOD recalled stop-point actions more accurately than did poor SOD participants. The main effect of learning condition, *F*(1, 73) = 0.05, *p* = 0.82, *η* ^*2*^ < 0.01, the main effect of gender, *F*(1,73) = 0.02, *p* = 0.90, *η* ^*2*^ < 0.01, and the interaction of SOD and learning condition were not significant, *F*(1, 73) = 1.17, *p* = 0.28, *η* ^*2*^ < 0.01.

### Direction estimates

**Fig. 7:**
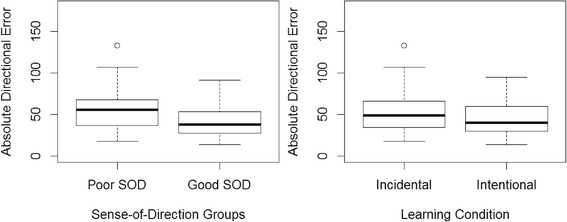
Absolute directional error by SOD group and learning condition. *Center* of the box represents the mean, the *top* and *bottom* of the box indicate the first and third quartile, the *whiskers* indicate a 95% confidence interval, and the *circles* outside the whiskers represent outliers

Mean absolute directional error was 50° (*SD* = 24°), which is substantial but still significantly better than chance of 90°, *t*(77) = –15.01, *p* < 0.001. A 2 SOD group (poor, good) by 2 learning condition (incidental, intentional) ANOVA found a significant main effect of SOD group, *F*(1, 73) = 76.07, *p* < 0.05, *η* ^*2*^ = 0.10. As suggested by Fig. 7, good SOD participants pointed to non-visible stop-points more accurately than did poor SOD participants. The main effect of learning condition, *F*(1, 73) = 2.28, *p* = 0.14, *η* ^*2*^ = 0.03, the main effect of gender, *F*(1,73) = 2.60, *p* = 0.11, *η* ^*2*^ = 0.01, and the interaction of SOD and learning condition were not significant, *F*(1, 73) = 0.22, *p* = 0.64, *η* ^*2*^ = 0.02.

### Distance estimates

**Fig. 8:**
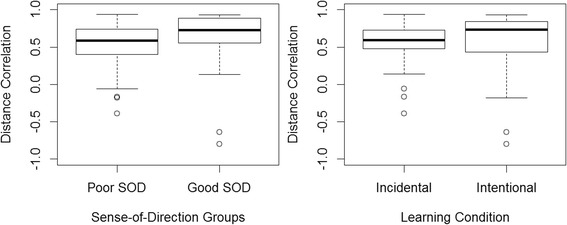
Distance correlation by SOD group and learning condition. *Center* of the box represents the mean, the *top* and *bottom* of the box indicate the first and third quartile, the *whiskers* indicate a 95% confidence interval, and the *circles* outside the whiskers represent outliers

Errors in distance estimation were calculated by correlating each participant’s set of estimated distances with the set of correct distances. These distance correlations express relative estimation accuracy across the set of distances; they are scale-independent, reflecting nothing about absolute overestimation or underestimation (Montello (1991) argues for the ambiguity of interpreting absolute distance estimation accuracy). Distance correlations were then converted using the Fisher r-to-z transformation before analysis. The mean distance correlation was *r* = 0.63 (*SD* = 0.49), only moderate but clearly significantly better than chance of *r* = 0, *t*(77) = 12.21, *p* < 0.001. The main effect of SOD, *F*(1, 73) = 2.47, *p* = 0.12, *η* ^*2*^ = 0.04, learning condition, *F*(1, 73) = 0.31, *p* = 0.58, *η* ^*2*^ = 0.01, the main effect of gender, *F*(1,73) = 0.03, *p* = 0.86, *η* ^*2*^ < 0.01, and the interaction of SOD and learning condition were not significant, *F*(1, 73) < 0.01, *p* = 0.99, *η* ^*2*^ < 0.01 (Fig. 8).

## Discussion

From the beginning of human evolution through the present time, the ability to learn the spatial layout of novel environments has been a critical human skill. However, people vary substantially in how easily and accurately they acquire spatial knowledge of environments. Their spatial knowledge acquisition skill relates fairly strongly, both conceptually and empirically, to an individual-difference variable known as “sense-of-direction.” We designed the current experiment to investigate this relationship, specifically if it depends on whether participants learn spatial relationships intentionally or incidentally. Clearly, self-report SOD predicted spatial knowledge acquisition virtually the same whether participants learned intentionally or incidentally—the two factors did not interact. Participants with a good SOD learned more accurate spatial knowledge than those reporting a poor SOD for landmark familiarity, route ordering, route actions, and direction estimation. In contrast, we found no main effect of learning intentionality, as participants who were intentionally instructed to learn spatial relationships did not acquire more accurate spatial knowledge than participants not instructed to learn. Presumably, under the incidental instructions, few if any participants consciously bothered to attend to or focus on spatial relationships experienced during travel.

One tenet of the alternative framework for spatial learning is that individuals with similar exposure to an environment will substantially differ in the amount and accuracy of spatial knowledge they acquire (Montello, 1998), due either to differences in relatively automatically applied abilities, consciously controlled strategies, or both. (While the authors of the dominant framework might have also agreed that individuals will differ in the accuracy of their spatial knowledge acquisition, their framework does not mention or imply this.) The empirical findings of Ishikawa and Montello (2006) strongly support the existence of substantial individual differences and our present results also reveal large variance among individuals, variance that correlates at least moderately with self-reported SOD. We took advantage of the well-known method of manipulating learning intentionality (Hasher & Zacks, 1979) to determine whether skill at spatial knowledge acquisition in the environment involves automatic or effortful processing. If variability in how well spatial knowledge is acquired expresses itself more after intentional than incidental learning, then we can conclude that this skill requires the conscious application of attention and knowledge processing. In contrast, if this variability expresses itself equally well under either set of instructions, we can conclude that this skill does not require conscious application of attention and knowledge processing.

Our results support the conclusion that the skill reflected by the construct of SOD does not require conscious application of attention and knowledge processing. Even when participants were misled about the purpose of the study, by being told to focus on architectural and natural features of the environment, they performed just as well as participants who knew they would be tested on the spatial configuration of the environment and were told to pay attention to it. Apparently, differences in spatial learning skills reflect implicit abilities and are expressed relatively automatically, without conscious effort. Our results provide no evidence that SOD reflects effortfully applied strategies or conscious attention to the spatial layout of the environment.

This conclusion is reminiscent of Neisser’s (1987) ideas about a “spatial module” for maintaining orientation and learning the environment. According to Neisser, this mind/brain system is specialized for processing spatial knowledge relevant to the space of locomotion, i.e., environmental space (Montello, 1993). It is a system that humans supposedly share with other animal species that extract spatial layout information from the operation of perception-action processes, integrating it to form mental representations of the environment, i.e., cognitive maps (see also Meilinger, 2008; Sholl, 1988; Sholl, Kenny, & DellaPorta, 2006; Yeap & Jefferies, 1999). Although our research does not speak to the issue of whether this system has the classic characteristics of modularity (Cheng & Newcombe, 2005), it is consistent with Neisser’s hypothesis that the system is sensitive to ongoing optical and proprioceptive information, and operates without conscious application.

A related possibility is that the implicit abilities that underlie SOD differences might stem from differing contributions from the components of working memory. Using dual-task designs, multiple studies have found that visual and spatial working memory are involved in spatial knowledge acquisition, the use of spatial knowledge, or both. Visuospatial and central executive working memory were found to be more involved in using, than developing, mental representations of an environment (Brunyé & Taylor, 2008). Visual and spatial working memory (especially the latter) were involved in encoding of route and survey knowledge (Labate et al., 2014; Van Doorn & Blokland, 2014), and switching perspectives (route or survey) between learning and testing (Meneghetti, Labate, Pazzaglia, Hamilton, & Gyselinck, 2016). Several studies have examined how differences in SOD are related to differences in the use of working memory. For tasks involving route knowledge, individuals with a good SOD relied more heavily on visuospatial than verbal working memory, while those with a poor SOD relied more heavily on verbal than visuospatial working memory (Baldwin & Reagan, 2009). Findings by Wen and colleagues extended this work to include landmark and survey knowledge. Individuals with a good SOD encoded landmark and route information using verbal and spatial working memory (Wen et al., 2011) and then integrated that egocentric (or body-centered) survey knowledge into allocentric (or environment-centered) survey knowledge using all three components of working memory (Wen, Ishikawa, & Sato, 2013). In contrast, individuals with a poor SOD encoded landmark information using verbal working memory, encoded route information using visual working memory (Wen et al., 2011), and then are unable to acquire accurate survey knowledge (Wen et al., 2013). As these studies indicate, good SOD participants may have performed better in our tasks, regardless of intentionality, due to the components of working memory that were involved in their encoding and processing of spatial information.

The current experiment apparently failed to fully replicate a previous finding by Van Asselen et al. (2006) of better spatial knowledge acquisition after intentional learning compared to incidental learning. Specifically, neither of us found a difference in landmark recognition and ordering between incidental and intentional learners, but van Asselen et al. reported that intentional learners performed survey spatial tasks more accurately. In fact, their survey tasks consisted of route-drawing on a detailed base map, route reversal, and route-distance estimation, none of which are clearly survey tasks. But one might still expect from van Asselen et al.’s results that our survey tasks would reveal the effects of learning condition (especially since our study was adequately powered to find van Asselen et al.’s large learning condition differences in survey tasks). However, there are several differences between our methods and theirs. Our route was outdoors and more than five times the length of van Asselen et al.’s route; their indoor route had the normal restricted vistas found in buildings. Also, their participants performed a 20-min distractor task before doing the route tasks. But we believe the most telling difference between our methods was the way we implemented our incidental learning conditions. Van Asselen et al. told their participants that a scheduling error had been made; they then walked along the test route ostensibly to reach the actual study room. Being led to a destination, without any reason to pay attention to the environment, their incidental participants could simply ignore their surrounds as they were walking. That is, their condition may well have been incidental to all types of route and environmental information, spatial and otherwise. In contrast, we did not claim a mistake but asked our participants to pay attention to the architectural appearance and design of the neighborhood, and specifically to “salient features.” Our condition was meant to be incidental to spatial information but not to the general appearance of the environment, which we intended as a more precise test of whether performance differences as a result of self-report SOD depend on whether people are trying to acquire spatial information or not. These differences may have contributed to the non-significant learning condition effects we found in our survey tasks (as our study was underpowered to detect small learning condition effects). Replicating our study with more thoroughly “incidental” procedures would be informative about the role of different types of attentional focus during environmental learning.

An important caveat of research designs that manipulate intention to learn (Chrastil & Warren, 2012, 2013) concerns the effectiveness of the manipulation. There is no guarantee that some or all of the incidental participants actually did ignore spatial information, nor that some or all of the intentional participants actually did attend to it. This caveat poses an important potential threat to the validity of our conclusions. It may be that SOD relates to one’s “natural” tendency to think about spatial properties, even when no one has told you to do so and there is no apparent need to do so (e.g., when you know a researcher will lead you back). This is consistent with the possibility that participants in the current experiment with a good SOD attended to spatial properties even in the incidental condition. But this caveat only poses a full threat to the validity of our conclusions if it is also true that people with a poor SOD have an equally strong natural tendency to ignore spatial properties when they are requested to attend to them. Another possibility is that participants might have been alerted to the spatial nature of the experiment by completing the prescreening; however, we think this possibility is unlikely. Both the prescreening (which included extra familiarity questions so as to avoid revealing the experiment location) and main experiment were framed to participants as an architectural study, and participants were never informed of the prescreening selection criteria. There was typically two weeks to a full month between the prescreening and experiment. No participants in the incidental learning condition told the researcher or revealed on the questionnaires that they knew the experiment was about learning the spatial layout of the environment. While it is possible that participants were aware of the spatial nature of the experiment, it is unlikely.

In the everyday context where no one tells you to attend to spatial properties (i.e., incidental learning), it is appealing to explain individual differences in maintaining orientation and acquiring spatial knowledge as resulting from people’s tendencies to focus on spatial properties or not, rather than their abilities as such. Such an explanation in the everyday context is plausible, in our view. But in the context of a behavioral-science experiment that randomly assigns participants to receive incidental or intentional instructions, we find this explanation to be much less plausible. Instead, we find it more likely that at least most of the participants in our two experimental groups differed substantially in their tendency to pay attention to spatial properties, in accordance with the instructions they received, indicating that differences in SOD do not merely reflect different tendencies to focus on spatial properties.

Even if differences in people’s SOD are relatively automatically expressed and not dependent on conscious effort, it is important to emphasize that this does not mean that environmental spatial skills cannot be improved through training, including training to apply particular learning strategies explicitly. Even innate traits can be modified by experience. Variations in body mass and hair color have unambiguously strong innate causes, but diet, exercise, and hair dye prove that genetics (let alone automaticity) is not destiny in any straightforward way. Indeed, work such as that by Cornell, Heth, and Rowat (1992) proves that environmental spatial knowledge can be improved by consciously applying learned strategies of spatial orientation.

Our results provide further support for the validity of self-reported SOD as a measure of spatial cognitive skills in the environment (Hegarty et al., 2002; Sholl et al., 2006). A novel result of our research is the substantial differences we found between the SOD groups for landmark familiarity and route knowledge. In previous work, SOD differences are typically small or non-existent for measures of landmark and route knowledge (Ishikawa & Montello, 2006). In fact, many studies of SOD do not assess landmark or route knowledge (e.g., Cornell, Sorenson, & Mio, 2003; Fields & Shelton, 2006; Hegarty et al., 2006; Montello & Pick, 1993; Schinazi et al., 2013). This is probably done either because the researchers think it will reveal no interesting relationships or because their experimental designs require participants to have a certain minimal level of landmark and route knowledge in order to assess survey knowledge. But the current research highlights that SOD differences can reveal themselves in less sophisticated forms of spatial knowledge, and that researchers should not assume that all participants have equal levels of landmark and route knowledge after similar exposure to an environment. It is likely that learning landmark identities and spatial relations along routes is simply much easier, even if it is not strictly required as a precursor for survey knowledge. In many studies, researchers will encounter ceiling effects in the acquisition of these types of knowledge and thus no relation to SOD. For example, participants in the study by Ishikawa and Montello (2006) completely accurately recalled and placed four named landmarks in order, on each of two routes, and even participants with poor SOD estimated the lengths of route segments after only one learning trial with a correlation of over 0.9 with the actual segment lengths. We also note that in studies like Ishikawa and Montello, landmarks are verbally labeled for participants, which may be less challenging because it offloads some spatial processing onto the verbal domain. This idea should be further explored empirically.

## Conclusions

By mimicking the experience of traveling to an unfamiliar destination while being led by someone familiar with the environment and then needing to return home unaided, the current experiment investigated the relationship between SOD and intentionality to learn the spatial relationships within a novel neighborhood. Across nearly all measures of spatial knowledge, individuals with a good SOD acquired spatial knowledge significantly more accurately than individuals with a poor SOD; whereas, intentionality to learn showed nearly no accuracy differences in the spatial knowledge acquired. This suggests that your SOD abilities are most predictive of your success in returning home unaided, not your attention to the spatial properties of the environment while traveling to your destination. These results are consistent with the idea that individual differences in environmental spatial learning reflect implicit abilities that are expressed relatively automatically. It does not necessarily suggest anything about the source of these abilities, however. Our findings have implications for research on the training of environmental spatial skills, as training programs or other interventions could be effective in teaching individuals how to successfully implement spatial strategies, but also, how to identify situations in which they need spatial strategies. Paying attention to spatial features, landmarks, and orientations is not the default mode for individuals with a poor SOD, as the current research has revealed; therefore, future work should investigate methods that could assist poor SOD individuals with attending to spatial features.

## Abbreviations

Est: Estimate
GPS: Global Positioning System
SBSOD: Santa Barbara Sense-of-Direction scale
SD: Standard deviation
SE: Standard error
SOD: Sense-of-direction
UCSB: University of California-Santa Barbara
